# TPPU Pre-Treatment Rescues Dendritic Spine Loss and Alleviates Depressive Behaviours during the Latent Period in the Lithium Chloride-Pilocarpine-Induced Status Epilepticus Rat Model

**DOI:** 10.3390/brainsci11111465

**Published:** 2021-11-05

**Authors:** Weifeng Peng, Yijun Shen, Qiang Wang, Jing Ding, Xin Wang

**Affiliations:** 1 Department of Neurology, Zhongshan Hospital, Fudan University, Fenglin Road, Shanghai 200032, China; peng.weifeng@zs-hospital.sh.cn (W.P.); shen.yijun1@zs-hospital.sh.cn (Y.S.); wang.qiang2@zs-hospital.sh.cn (Q.W.); 2 Department of The State Key Laboratory of Medical Neurobiology and MOE Frontiers Center for Brain Science, Institutes of Brain Science, Fudan University, Shanghai 200032, China

**Keywords:** epileptogenesis, depression, comorbidity, soluble epoxide hydrolase, inflammation, dendritic spine

## Abstract

Epileptogenesis may be responsible for both of recurrent seizures and comorbid depression in epilepsy. Disease-modifying treatments targeting the latent period before spontaneous recurrent seizures may contribute to the remission of seizures and comorbid depression. We hypothesized that pre-treatment with 1-trifluoromethoxyphenyl-3-(1-propionylpiperidin-4-yl) urea (TPPU), a soluble epoxide hydrolase (sEH) inhibitor, which has anti-inflammatory and neuroprotective effects might rescue status epilepticus (SE)-induced dendritic spine loss and alleviate depressive behaviours. Rats were either pre-treated with TPPU (0.1 mg/kg/d) intragastrically or with vehicle (40% polyethylene glycol 400) from 7 days before to 7 days after SE that was induced with lithium chloride and pilocarpine intraperitoneally. Rats in the Control group were given saline instead. The forced swim test (FST) was performed on the 8th day after SE to evaluate the depression-like behaviours in rats. The results showed that seizures severity during SE was significantly decreased, and the immobility time during FST was significantly increased through TPPU pre-treatment. Moreover, pre-treatment with TPPU attenuated inflammations including microglial gliosis and the level of proinflammatory cytokine IL-1β in the hippocampus; in addition, neuronal and dendritic spine loss in the subfields of hippocampus was selectively rescued, and the expression of NR1 subunit of N-methyl-D-aspartate (NMDA) receptor, ERK1/2, CREB, and their phosphorylated forms involved in the dendritic spine development were all significantly increased. We concluded that pre-treatment with TPPU attenuated seizures severity during SE and depressive behaviours during the period of epileptogenesis probably by rescuing dendritic spine loss in the hippocampus.

## 1. Introduction

Epilepsy is a chronic brain disease characterized by abnormal hyperexcitability of neurons that may cause structural and functional network damages of the brain [1]. About 30–40% of patients with epilepsy are refractory to anti-seizure medications although there are increasing new development of drugs [2]. Moreover, 25–75% of patients with epilepsy have neuropsychiatric comorbidities such as depression which is much more higher than non-epileptic controls [3]. The overlapping functional network between epilepsy and depression indicates the process of epileptogenesis may be responsible for the occurrence of recurrent seizures and comorbid depression in epilepsy [3,4].

The term epileptogenesis refers to the process by which a normal brain becomes epileptic. The experimental status epilepticus (SE) animal models induced by chemicals or continuous electrical stimulations usually have a transient episode of SE manifested motor seizures and then followed a seizure-free “latent period” of one to several weeks, after that, spontaneous seizures emerge [5]. Epileptogenesis might occur in the “latent period”, which mimics the human temporal lobe epilepsy, for example, SE caused by acute brain injury or febrile convulsions in young children followed by recurrent seizures in their later lives [6]. Therefore, disease-modifying drugs targeting the latent period after SE may contribute to the remission of seizures and comorbidities.

Inflammations were found to be involved in the epileptogenesis in many types of epilepsy animal models [7]. Anti-inflammatory treatments improved both seizures and associated behavioral comorbidities including depression in these models; simultaneously, the level of plasma IL-1β and gliosis in specific limbic regions of the brain were reduced [8,9]. The TPPU [1-trifluoromethoxyphenyl-3-(1-propionylpiperidin-4-yl) urea], an inhibitor of soluble epoxide hydrolase (sEH), is a lipid-soluble chemical compound which can pass through blood-brain barrier and take anti-inflammatory effects in the central nervous system, and it has high oral availability and excellent pharmacokinetics [10,11]. Studies demonstrated that TPPU had anti-seizure and anti-depressant effects, which might take effects through increasing the level of metabolic substrates such as epoxyeicosatrienoid acids (EETs) [10,12,13]. We hypothesized that pre-treatment with TPPU targeting the latent period that represents the epileptogenesis process might alleviate seizures severity and comorbid depression.

In previous studies, SE-induced loss or remodeling of dendritic spines was observed and proposed to be a pathological reorganization of synaptic network correlated with neuronal hyperexcitability [14,15]. The dendritic spine is a protrusion located at postsynaptic area that opposed to the presynaptic terminals of most excitatory synapses of principle neurons in the brain [16]. Particularly, the new spine growth is vital for maintaining synaptic stability and participating in regulating moods, learning, and memory functions [17]. Glutamate ionotropic N-methyl-D-aspartate (NMDA) receptor which mediates the activity of excitatory neurotransmitters and influx of calcium is richly expressed on postsynaptic density (PSD) of dendritic spine [18]. Extracellular regulated kinase (ERK1/2) is involved in Mitogen-Activated Protein Kinase (MAPK) downstream signaling that mediated by NMDA receptor [19], which plays an important role in relaying signal from the stimulated spines to nucleus [20]. The cyclic adenosine monophosphate-response element binding protein (CREB) is the transcription factor regulated by ERK1/2, which promotes gene transcription and takes neuroprotective effect [21]. The NMDA receptor/ERK1/2/CREB pathway is crucial for dendritic spine development and functional maintenance. Besides, Moda-Sava et al. demonstrated that the branch-specific elimination of postsynaptic dendritic spines of prefrontal cortex was associated with depression-related behavior in chronic stress models [22]. Therefore, SE-induced spine loss might participate in the epileptogenesis and contribute to comorbid depression in epilepsy. In this study, we tried to explore whether depression occurred during the latent period that represents the process of epileptogenesis and pre-treatment with TPPU could attenuate seizure severity during SE, rescue SE-induced dendritic spine loss, and alleviate the comorbid depression in the LiCl-pilocarpine-induced SE rat model.

## 2. Methods

### 2.1. Experimental Animals

Male adult Sprague–Dawley rats aged 6–8 weeks and weighing 200–250 g (supplied by Shanghai Charles River Laboratory) were used in this study. The environment for rats was set at 22–25 °C and under a 12-h day_night cycle. The rats were free access to food and water. The experiment was done in accordance with the guidelines of the National Institutes of Health and approved by the Committee of Animal Care and Use in Zhongshan Hospital of Fudan University. Efforts were made to minimise animal suffering and the number of animals used.

### 2.2. Establishment of the LiCl-Pilocarpine-Induced SE Rat Model and TPPU Treatment Paradigm

As described previously [23], rats were given LiCl (127 mg/kg, dissolved in water, Sigma, St. Louis, MO, USA) by intraperitoneal (I.P.) injections. Scopolamine methyl bromide (1 mg/kg, Sigma-Aldrich, Sacramento, CA, USA) and pilocarpine (40 mg/kg, Sigma-Aldrich, USA) were I.P. given sequentially 24 h later with 30 min interval. A modified Racine scale was used to evaluate seizure severity [24]. The criterion of SE in this study was set as recurrent seizures greater than or equal to Racine stage 3 and sustaining for 30 min. Rats that met SE criterion were treated with diazepam (10 mg/kg, Tianjin, China) to terminate seizures. Rats were then kept in a transparent cage and monitored with a video surveillance system (a CCD camera, JVC, Yokohama, Japan) to observe the recurrent seizures.

**Figure 1:**
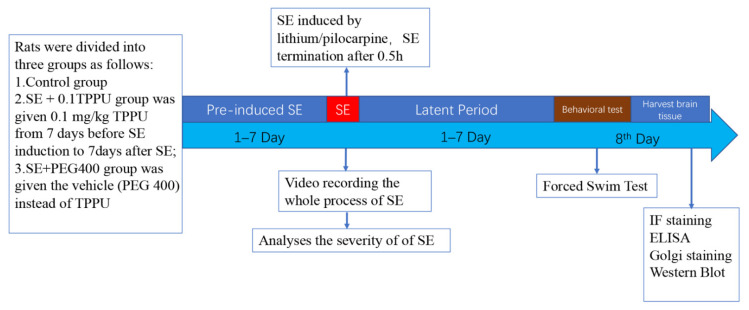
Schematic illustration of the protocol used in the experiment. TPPU or vehicle treatment paradigm: TPPU or vehicle was given intra-gastrically from 7 days before SE induction to 7 days after SE, and the forced swim test was performed on the 8th day after TPPU treatment. SE, status epilepticus; TPPU, 1-trifluoromethoxyphenyl-3-(1-propionylpiperidin-4-yl) urea; IF, immunofluorescent; ELISA, enzyme-linked immunosorbent assay.

TPPU was produced by the lab of Prof. Bruce D. Hammock at the University of California Davis. According to the pharmacological characteristics and half-life elimination [24,25], TPPU was dissolved in a saline solution containing 40% polyethylene glycol 400 (PEG 400), and the volumes of 1–1.5 mL TPPU (0.1 mg/kg/d, abbreviated as 0.1TPPU) were administered by gastric gavage at 8 am every morning from 7 days before to 7 days after SE induction (see Figure 1). Based on given TPPU or vehicle, the rats were randomly divided into the following groups: (1) the SE+0.1TPPU group was given 0.1 mg/kg TPPU; (2) the SE+PEG 400 group was given the vehicle (PEG 400) instead of TPPU; (3) the Control group was given LiCl and PEG 400, but not pilocarpine.

### 2.3. Forced Swim Test (FST)

The FST was performed using the same procedure of our previous work [23]. The longer immobility time (IMT) in swimming behaviours is indicative of the behaviour of despair [25]. FST was performed on the 8th day after SE (see Figure 1). The rats were observed for 2 h to make sure that no seizures occurred before FST to avoid the immediate influence of seizures on the outcome of behavioural assay. After FST, the rats were sacrificed, and the rats’ brains were processed according to the subsequent procedures. Every fourth brain was used to conduct immunofluorescent staining, Golgi staining, and protein extraction for ELISA and western-blot protein analysis.

### 2.4. Immunofluorescent Staining

After behavioural tests, the rats were deeply anesthetized with 10% chloral hydrate (3 mL/kg, I.P.) and perfused trans-cardinally with 4 °C saline, followed by 4% paraformaldehyde in phosphate-buffered saline (PBS) (10 mM, pH 7.4). Then rats were decapitated, and their brains were removed and put into 4% paraformaldehyde at 4 °C for 24 h, and then were shifted to 20–30% sucrose in 0.1 M PBS at 4 °C until sinking. The following procedures were conducted: (1) coronal sections (10 μm) through the dorsal hippocampus were prepared using a freezing microtome (CM1950, Leica, Heidelberg, Germany); (2) sections through the hippocampus was selected from each rat (Bregma −4.68 to −4.20 mm); (3) sections were incubated with the primary antibody at 4 °C for 24 h. The primary antibodies used in this study were as follows: the rabbit monoclonal anti-glial fibrillary acidic protein (GFAP) primary antibody (1:1000, Millipore), the rabbit anti-ionized calcium binding adapter molecule 1 specific protein (Iba-1, 1:200, Abcam, Cambridge, UK), and the mouse anti-neuronal specific nuclear protein (NeuN, 1:600, Millipore, Billerica, MA, USA); (4) after washing for 3 times, sections were incubated with the secondary antibodies (anti-rabbit, Alexa 546; anti-mouse, Alexa 488, Molecular Probes, Cambridge, England) for 1 hr at room temperature; (5) photomicrographs of CA1, CA3, and dentate gyrus (DG) subfields of the hippocampus (Hip) were taken at 20× magnification from the sections under a fluorescent microscope (Olympus/BX51, Tokyo, Japan).

### 2.5. Golgi Staining

Four rats of each group were processed with the Golgi-Cox impregnation technique using the commercially available Hito Golgi-Cox OptimStain kit (Hitobiotec Corp, Kingsport, TE, USA). The impregnation solution was made by mixing liquid A and liquid B 1:1 for 24 h and then the supernatant was taken for use. Brain tissues of the rats were briefly rinsed twice in double distilled water (dd H_2_O), and then soaked into the impregnation solution overnight at 25 °C. The next day, tissues were incubated in a fresh impregnation solution, stored in the dark at 25 °C for 2 wks, and then transferred in solution C in the dark at 25 °C for 1 day. Thereafter, tissues were placed into a fresh solution C in the dark at 25 °C for 6 days. Then, solution C was removed, and samples were stored at 4 °C in the dark overnight. Tissues were embedded in OCT (O.C.T. Compound SAKURA, Torrance, CA, America) and sectioned with a vibratome in the coronal plane at 100–250 mm thickness, and then transferred to dd H_2_O and washed for several times. The next day, sections were put into a mixture of solutions D and E for10 min at 25 °C. After washing in dd H_2_O twice with 5 min each time, sections were dehydrated in 50%, 70% and 95% ethanol for 5 min, respectively, and then transferred into absolute ethanol (5 min) and transparent in xylene twice with 5 min each time. Lastly, sections were examined and imaged with an Olympic optical microscope.

### 2.6. Tissue Preparation and Protein Extraction

The rats were euthanized by cervical dislocation after being deeply anesthetized with 4% chloral hydrate, and then the brains of the rats were quickly removed from the skull and put into ice-cold PBS. The tissue of hippocampus was dissected out for protein extraction. The process of tissue protein extraction was based on the standard procedure of Beyotime reagent kit (Beyotime Institute of Biotechnology, Shanghai, China).

### 2.7. Enzyme-Linked Immunosorbent Assay (ELISA)

As described previously [13], the Luminex kit (Youningwei, Shanghai, China) was used to measure cytokines (TNF-α, IL-1β, and IL-6) in the hippocampus of rats. The procedures were as follows: (1) adding 50 μL of the standard or samples to each well, (2) adding 50 μL of diluted microparticle cocktail to each well, (3) incubating for 2 h at room temperature (RT) on a shaker at 800 rpm, (4) removing the liquid from each well and filling the well with 100 μL wash buffer, performing the wash three times, (5) adding 50 μL of diluted biotin-antibody cocktail to each well, covering, and incubating for 1 hr at RT on the shaker at 800 rpm, (6) repeating 3 times washing as before, (7) adding 50 μL of diluted streptavidin-PE to each well and incubating for 30 min at RT on the shaker at 800 rpm, (8) repeating the wash 3 times, (9) adding 100 μL of wash buffer to each well, covering, and incubating for 2 min at RT on the shaker at 800 rpm. A Luminex analyser was used to read the results.

### 2.8. Western Blot Analysis

The procedures were as follows: (1) protein extracts were separated by sodium dodecyl sulphate-polyacrylamide gel electrophoresis (SDS-PAGE) and then transferred to cellulose acetate membranes; (2) the membranes were blocked using goat serum and incubated with primary antibodies including rabbit anti-CREB (43 kDa, 1:1000, CST, Boston, MA, USA), rabbit anti-CREB-phospho Ser133 (43 kDa, 1:1000, CST, Boston, MA, USA), rabbit anti-ERK1/2 (43 kDa, 1:1000, CST, Boston, MA, USA), and rabbit anti-phospho-p44/42 ERK1/2 (Thr202/Tyr204) (42/44 kDa, 1:1000, CST), rabbit anti-NMDAR1 (120 kDa, 1:1000, CST, Boston, MA, USA), and rabbit anti-NMDAR1-phosphoS889 (120 kDa, 1:1000, CST, Boston, MA, USA) at 4 °C for 24 h; (3) the rabbit anti-β-actin primary antibody (40 kDa, 1:1000, Beyotime, Shanghai, China) was used as an internal reference; (4) after 24 h later, the membrane was incubated with the goat anti-rabbit IgG secondary antibody (1:1000, Beyotime, Shanghai, China) for 2 h at RT; (5) the Tanon Image software (version 4100, Shanghai, China) was used to analyse the bands of target proteins. The optical density (OD) value of each sample was normalised by the corresponding amount of β-actin.

### 2.9. Statistical Analysis

The Graphpad Prism 8 software was used to conduct the statistical analysis in this study. All the data in this study were conducted using Shapiro Wilk test and it’s verified to conform to normal distribution. Therefore, comparisons between groups were performed using the Student t test, one-way analysis of variance (ANOVA) test, or two-way ANOVA test. A post-hoc Tukey test was adopted for comparisons between two groups in the one-way ANOVA test. A *p*-value of less than 0.05 was statistically significant. The data were expressed as mean ± standard error of the mean (SEM).

## 3. Results

### 3.1. Pre-Treatment with TPPU Decreased the Seizure Severity during SE

Thirty-six rats were randomly divided into three groups: the Control group, the SE+PEG400 group, and the SE+0.1TPPU group (*n* = 12, respectively, in every group). The sample size was determined by the statistical calculator in which the difference of IMT equal to 10 s was set as the significance of group difference based on our previous work [23]. The mortality was 3/12 and 2/12 in the SE+PEG400 and SE+0.1TPPU groups after SE induction, respectively. Another three and two rats were added into these two groups according to the same experimental paradigm to make the number of rats equal in the three groups.

**Figure 2:**
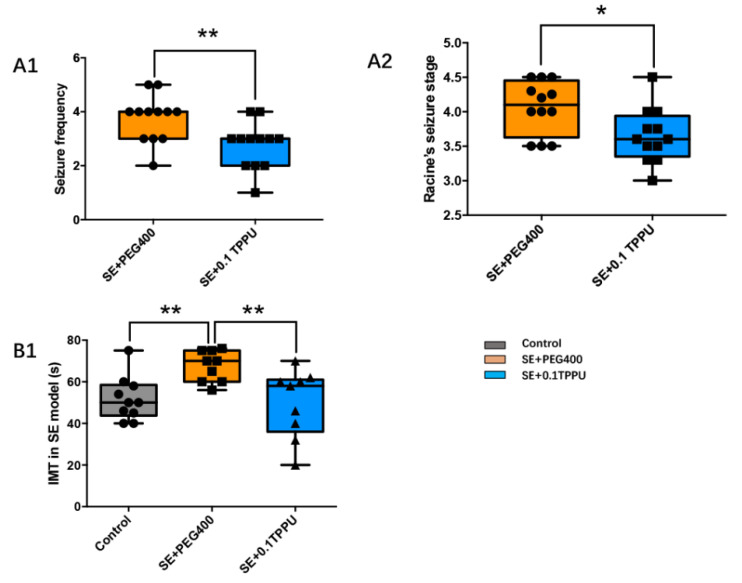
The seizure frequency (**A1**) and Racine’s seizure grade (**A2**) during status epilepticus were decreased significantly in the SE+0.1TPPU group compared with SE+PEG400 group. (**B**) The immobility time (IMT) was significantly increased in the SE+PEG400 group compared with the Control group, which was significantly decreased after TPPU treatment in the SE+0.1TPPU group compared with the SE+PEG400 group. (Figure 2A1, student *t*-test, Figure 2A,B, one-way ANOVA test, * *p* < 0.05, ** *p* < 0.01, *n* = 12). SE, status epilepticus; IMT, the immobility time.

In the SE+0.1TPPU group that was pre-treated with TPPU, the seizure frequency and Racine’s seizure grade during SE were decreased significantly as compared with the SE+PEG400 group (Figure 2A1,A2, * *p* < 0.05, ** *p* < 0.01, *n* = 12).

### 3.2. Pre-Treatment with TPPU Attenuated the Depressive Behaviour of Despair 7 Days after SE Induction

The depressive behaviour in rats was evaluated by FST on the 8th day after SE induction. No seizures were observed during the two hours before FST. The result showed that the IMT, an indicator for despair, was significantly increased in the SE+PEG400 group compared with the Control group, which was significantly decreased in the SE+0.1TPPU group that was pre-treated with TPPU compared with the SE+PEG400 group. (Figure 2B, ** *p* < 0.01, *n* = 12).

### 3.3. Pre-Treatment with TPPU Alleviated Inflammations in the Hippocampus of LiCl-Pilocarpine-Induced SE Rat Model

**Figure 3:**
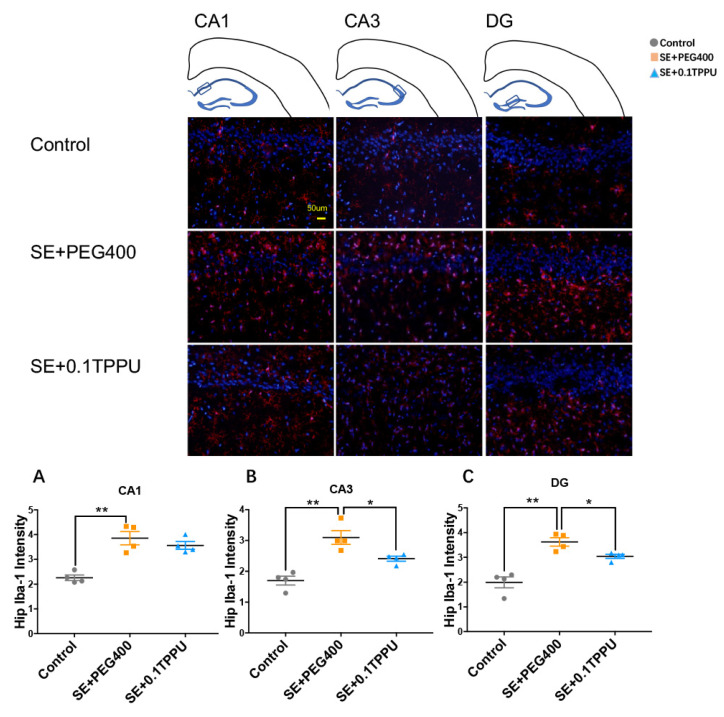
(**A**) The Iba-1 intensity in CA1 subarea was significantly increased in SE+PEG400 group compared with Control group. (**B**,**C**) The Iba-1 intensity in CA3 and DG subareas was significantly increased in SE+PEG400 group compared with Control group, which was significantly decreased in SE+0.1TPPU group (Figure 3A–C, one-way ANOVA test, * *p* < 0.05, ** *p* < 0.01 *n* = 4). CA, Cornu Ammonis; DG, dentate gyrus; Hip, hippocampus. Iba-1, ionized calcium binding adaptor molecule-1.

**Figure 4:**
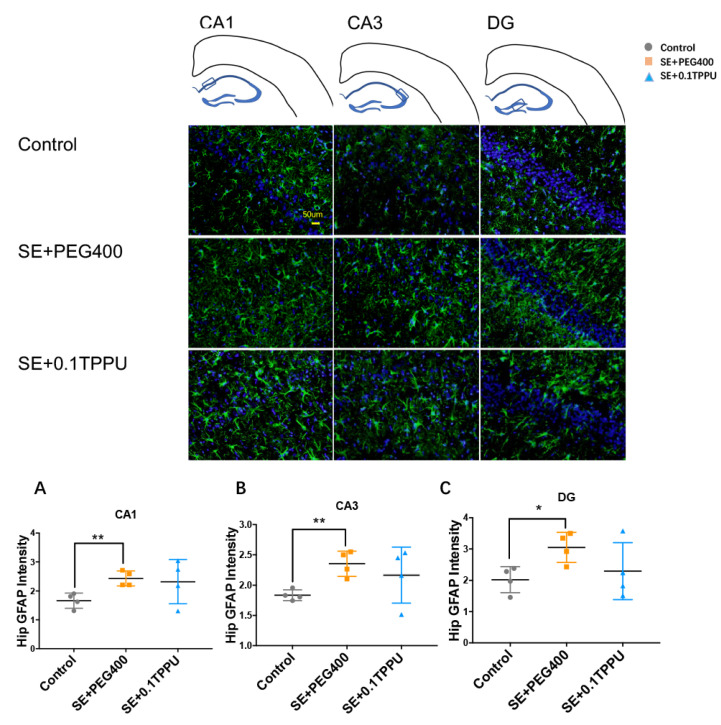
The fluorescence intensity of GFAP in CA1 subarea (**A**), CA3 subarea (**B**), and DG subarea (**C**) was significantly increased in SE+PEG400 group compared with Control group (Figure 4A–C**,** one-way ANOVA test, *n* = 4 in every group, * *p* < 0.05, ** *p* < 0.01). CA, Cornu Ammonis; DG, dentate gyrus; Hip, hippocampus. GFAP, glial fibrillary acidic protein.

Iba-1 was used to mark the expression and activity of microglia in the hippocampus and of rats. The number of Iba-1 positive microglial cells was significantly greater in the CA1, CA3, and DG subareas of hippocampus of the SE+PEG400 group than the Control group, and TPPU pre-treatment significantly attenuated microglial activation in the CA3 and DG subareas of hippocampus in the SE+0.1TPPU group compared with the SE+PEG400 group (Figure 3A–C, *n* = 4 in every group, * *p* < 0.05, ** *p* < 0.01). GFAP was stained to mark the expression and activity of astrocytes in the hippocampus of rats. The number of GFAP positive astrocytes was significantly increased in the CA1, CA3, and DG subareas of hippocampus of the SE+PEG400 group compared with the Control group, however, there was no difference of astrocytic gliosis between the SE+0.1TPPU group and the SE+PEG 400 group (Figure 4A–C, *n* = 4 in every group, * *p* < 0.05, ** *p* < 0.01).

**Figure 5:**
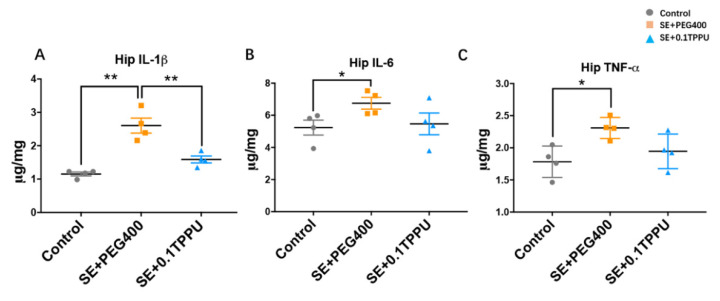
The levels of pro-inflammatory cytokines including IL-1β, IL-6, and TNF-α were significantly increased in the hippocampus and prefrontal cortex of the SE+PEG400 group compared with the Control group (**A**–**C**). The level of IL-1β was solely significantly attenuated in the hippocampus of SE+0.1TPPU group compared with the SE+PEG400 group (**A**). (Figure 5A–C**,** one-way ANOVA test, *n* = 4 in every group, * *p* < 0.05, ** *p* < 0.01). Hip, hippocampus. IL-1β, interleukin-1β; IL-6, Interleukin-6; TNF-α, tumor necrosis factor.

The levels of pro-inflammatory cytokines in the hippocampus of rats were determined by the ELISA method. The results showed that the levels of pro-inflammatory cytokines including IL-1β, IL-6, and TNF-α were significantly increased in the hippocampus of the SE+PEG400 group compared with the Control group, and the level of IL-1β was solely significantly attenuated in the hippocampus of SE+0.1TPPU group compared with the SE+PEG400 group (Figure 5A–C, *n* = 4 in every group, * *p* < 0.05, ** *p* < 0.01).

### 3.4. Pre-Treatment with TPPU Selectively Rescued Neuronal and Dendritic Spine Loss in the Hippocampus of LiCl-Pilocarpine-Induced SE Rat Model

**Figure 6:**
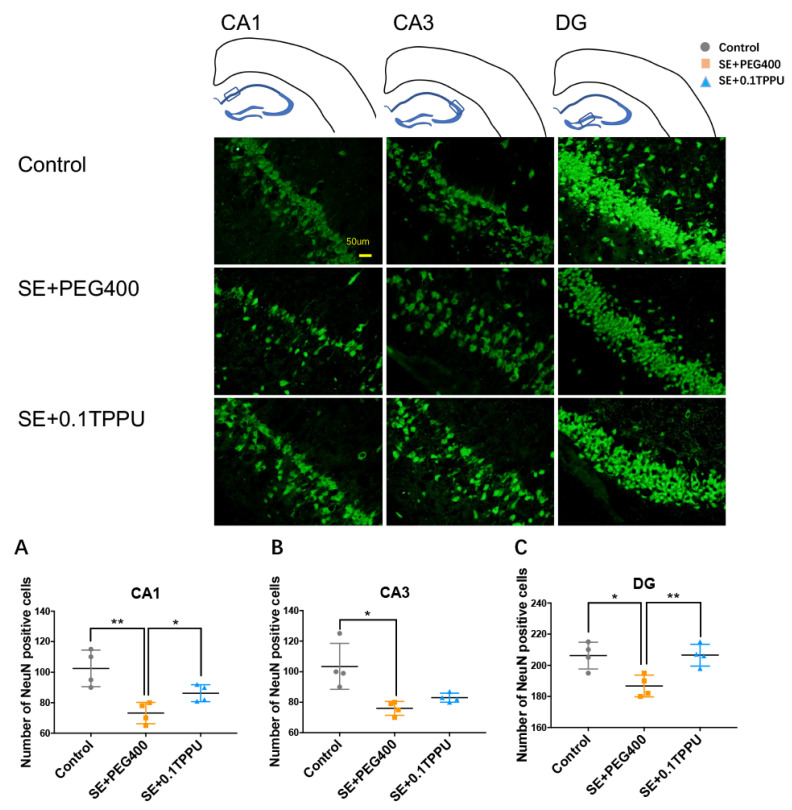
The number of NeuN positive neurons was significantly decreased in the CA1 subarea (**A**), CA3 subarea (**B**), and DG subarea (**C**) of the SE+PEG400 group compared with Control group, which was significantly increased in the CA1 (**A**) and DG (**C**) subareas of hippocampus after TPPU treatment in the SE+0.1TPPU group compared with the SE+PEG400 group. (Figure 6A–C, one-way ANOVA test, *n* = 4 in every group, * *p* < 0.05, ** *p* < 0.01). CA, Cornu Ammonis; DG, dentate gyrus; Hip, hippocampus; NeuN, neuronal nuclei.

The neuronal loss was measured by the NeuN staining. The results showed that the number of NeuN positive neurons was significantly decreased in the hippocampus of the SE+PEG400 group compared with Control group, which was significantly increased selectively in the CA1 and DG subareas of hippocampus after TPPU treatment in the SE+0.1TPPU group compared with the SE+PEG400 group (see the Figure 6A–C, *n* = 4 in every group, * *p* < 0.05, ** *p* < 0.01).

**Figure 7:**
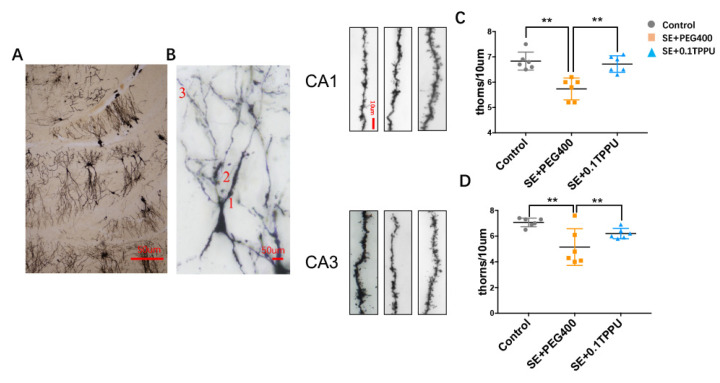
(**A**,**B**) The dendritic spines density of the third branch (×20 magnification in Figure 6A, ×40 magnification in Figure 6B) in the hippocampus. (**C**,**D**) The number of dendritic spines every 10 μm length of the third branch (×60 magnification) was significantly decreased selectively in the CA1 and CA3 subareas of hippocampus in the SE+PEG400 group compared with the Control group. TPPU pre-treatment significantly recovered the dendritic spines density in the SE+0.1TPPU group compared with the SE+PEG400 group. (Figure 7A,B, one-way ANOVA test, *n* = 4 in every group, ** *p* < 0.01. CA, Cornu Ammonis.CA1, CA3: The third branch of dendritic spines in the pyramidal neurons of CA1 and CA3 subareas were photographed and analysed.

The dendritic spines density of the third branch (Figure 7A,B) in the hippocampus was analysed. Each branch of dendritic spines was taken for 10 consecutive photographs and overlapped. Every 6 third branch of dendritic spines in each rat were selected for analysis. The number of dendritic spines every 10μm length of the third branch was significantly decreased selectively in the CA1 and CA3 subareas of hippocampus of the SE+PEG400 group compared with the Control group, and TPPU pre-treatment significantly recovered the dendritic spines density in the SE+0.1TPPU group compared with the SE+PEG400 group (Figure 7C,D, *n* = 4 in every group, * *p* < 0.05, ** *p* < 0.01).

### 3.5. TPPU Pre-Treatment Activated the NR1/ERK/CREB Pathway in the Hippocampus of LiCl-Pilocarpine-Induced SE Rat Model

**Figure 8:**
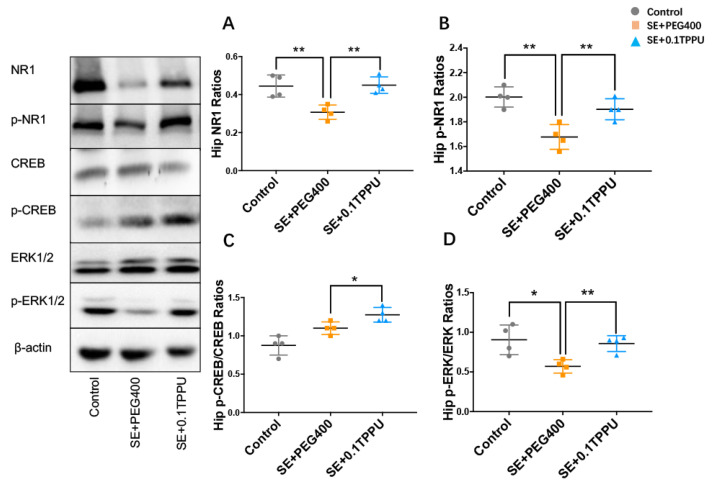
(**A**,**B**) The ratios of NR1 and p-NR1 subunits of NMDA receptor was significantly decreased in the hippocampus of the SE+PEG400 group compared with the Control group, which was significantly increased in the SE+0.1TPPU group compared with the SE+PEG400 group after TPPU treatment. (**C**,**D**) The ratios of p-ERK1/2/ERK1/2 and p-CREB/CREB in the hippocampus were significantly increased in the SE+0.1TPPU group compared with the SE+PEG400 group. (Figure 8A–D, one-way ANOVA test, *n* = 4 in every group, * *p* < 0.05 ** *p* < 0.01). The figure on the left showed the bands of NR1, p-NR1, CREB, p-CREB, ERK1/2, p-ERK1/2, and β-actin in the Control, SE+PEG400, and the SE+0.1TPPU groups measured by the western-blot method.Hip, hippocampus; NR1, N-methy1-D-aspartate 1 receptor; p-NR1, phospho(S889)-N-methy1-D-aspartate 1 receptor; CREB, cyclic adenosine monophosphate-response element binding protein; p-CREB, phosphor (Ser133)-cyclic adenosine monophosphate-response element binding protein; ERK1/2, extracellular regulated kinase; p-ERK1/2, phospho (Thr202/Tyr204)-p44/42 extracellular regulated kinase.

The expression and activities of the NR1 subunit of NMDA receptor, ERK1/2, and CREB were measured by the western blot analysis of the total proteins and their phosphorylated forms. The ratios of NR1 and p-NR1 subunits of NMDA receptor were significantly decreased in the hippocampus of the SE+PEG400 group compared with the Control group, which was significantly increased in the SE+0.1TPPU group compared with the SE+PEG400 group (Figure 8A,B, *n* = 4 in every group, * *p* < 0.05). The ratios of *p*-ERK1/2/ERK1/2 and p-CREB/CREB in the hippocampus were both significantly increased in the SE+0.1TPPU group compared with the SE+PEG400 group (Figure 8C,D, *n* = 4 in every group, * *p* < 0.05).

## 4. Discussion

In this study, we found that pre-treatment with TPPU from 7 days before to 7 days after SE alleviated seizure severity of SE and comorbid depressive behaviours during the period of epileptogenesis in the LiCl-pilocarpine-induced SE rat model. Meanwhile, the inflammations including the proinflammatory cytokine IL-1β and microgliosis were attenuated, dendritic spine loss was rescued, and the activities of NR1/ERK1/2/CREB pathway involved in spine development were activated in the hippocampus via TPPU pre-treatment.

Clinically, the prevalence of refractory TLE is as high as 30–40% and the mechanism of epileptogenesis in TLE is not clear yet [26,27]. The LiCl-pilocarpine-induced rat epilepsy model mimics the human TLE and it could be used to study the mechanism of epileptogenesis in TLE [6]. We determined 7 days after SE as the “latent period” of LiCl-pilocarpine-induced rat SE model in our study based on a previous research which demonstrated the “latent period” was about 7.2 ± 3.6 d [28]. On the 8th day after SE induction, the rats were evaluated by the FST and it indicated that the rats had depressive behaviours during the latent period before the occurrence of spontaneous seizures, which might be consistent with the clinical phenomenon that patients with newly-diagnosed or new-onset epilepsy had a high prevalence of comorbid depression, with 65% mild and 21% moderate to severe depression [29]. The finding of a large-scale population-based study that the severity of depression was positively correlated with the severity of epilepsy supported the process of epileptogenesis might be involved in the mechanism of epilepsy and the comorbidity of depression either [30]. Meanwhile, antidepressant therapies attenuated both depressive symptoms and seizure frequency concurrently [31].

The underlying pathological mechanisms of epileptogenesis are not clear yet. Hippocampal neuronal death, proliferation of astrocytes, and inflammations were observed in the hippocampal sclerosis of patients with temporal lobe epilepsy (TLE) and pilocarpine induced epilepsy rodent models [14,32]. In addition, Tang et al. found that neurons from the epileptic brain have a marked loss of dendritic spine [32], and the extent of dendritic spine loss was found to be correlated with the duration of the seizure disorder [33]. In the pilocarpine rodent epilepsy model, the time course of dendritic spine loss was maximal from 1 to 3 days after SE, partial recovery of spine density occurred 15 and 35 days later [33]. Our study was consistent with previous research that the dendritic spine density in the CA1 and CA3 subfields of hippocampus was selectively decreased on the 8th day after SE. Simultaneously, inflammatory parameters including proinflammatory cytokines, microglia, and astroglia were all enhanced significantly. We proposed that the dendritic spine loss in the period of epileptogenesis might be caused by the inflammations, which was also supported by the evidence that inflammations such as lipopolysaccharides exposure, viral immune activation, and prolonged exposure to inflammatory extracellular vesicles led to a significant decrease in dendritic spine density in hippocampal neurons and hippocampal slices in vivo and vitro [34,35,36].

TPPU is an inhibitor of sEH. Epoxygenated fatty acids (EpFAs), including epoxyeicosatrienoid acids (EETs), are endogenous substrates for sEH, which are hydrolysed by sEH and converted to their respective diols that take harmful effects in regulating inflammation [37,38]. Studies showed that the neuroprotective effect of TPPU is contributed by inhibiting the sEH enzyme activity thus increasing the levels of the metabolic substrates such as EETs and other EpFA that have pro-resolving and anti-inflammatory effects [39,40]. A study showed that combined injection of the sEH inhibitor with EETs into the brains of mice delayed the onset of pentylenetetrazol-induced seizures [41], and a previous study indicated that EETs activated K^+^ channels and had anti-inflammatory effects [42]. Ren et al. demonstrated that pre-treatment with TPPU prevented the onset of depression-like behaviours after inflammation or repeated social defeat stress [10]. Our study indicated that TPPU pre-treatment could attenuate seizure severity during SE and depressive behaviour during the latent period in the LiCl-pilocarpine-induced rat SE model.

In this study, TPPU pre-treatment not only resolved the inflammations but also rescued the dendritic spine loss in the hippocampus, and simultaneously increased the expression and activity of NR1/ERK1/2/CREB pathway in the LiCl-pilocarpine-induced rat SE model. There was a pronounced increase of IL-1β and microgliosis by TPPU pre-treatment, and the reason for this might be due to the significantly higher levels of IL-1β and activation of microglia than other proinflammatory factors during the epileptogenesis [43]. The NR1 subunit is obligatory for the composition of NMDA receptor complex [44], thus, the expression and activity of NR1 reflects the level and function of total NMDA receptor in the brain region. The NR1/ERK1/2/CREB pathway is important for dendritic spine development and function maintenance [20,21]. In consequence, we proposed that pre-treatment with TPPU might resolve the inflammations provoked by SE and then rescued the spine loss in the hippocampus of LiCl-pilocarpine-induced rat SE model, which was expected to be involved in the mechanism of anti-depressant effect of TPPU, as dendritic spine loss was responsible for depressive behaviours in the depression animal models [22].

There are some limitations in this study. The first is the relatively small size of rats in every part of experiment due to the different tissue processing procedures. Another one is that the spine loss may need to be further demonstrated by other methods in vivo and in vitro such as electron microscope or two-photon imaging in future.

## 5. Conclusions

In this study, we demonstrated that pre-treatment with TPPU reduced seizure severity during SE, rescued spine loss probably by taking anti-inflammatory effect, and then alleviated depressive behaviours during the latent period that represents the process of epiletogenesis. As there are no effective anti-epileptogenic medicines until now, our study indicates TPPU or other sEH inhibitors administered during the latent period might be potential disease-modifying medicines targeting epileptogenesis and warrant more detailed studies in the future.

## Data Availability

The raw data supporting the conclusions of this article will be made available by the authors, without undue reservation.
